# Why a New Research Agenda on Green Spaces and Health Is Needed in Latin America: Results of a Systematic Review

**DOI:** 10.3390/ijerph18115839

**Published:** 2021-05-29

**Authors:** David Rojas-Rueda, Elida Vaught, Daniel Buss

**Affiliations:** 1 Department of Environmental and Radiological Health Sciences, Colorado State University, 1601 Campus Delivery, Fort Collins, CO 80523, USA; 2 Pan American Health Organization, 525 23rd Street NW, Washington, DC 20037, USA; emottasousa@hotmail.com (E.V.); bussd@paho.org (D.B.)

**Keywords:** green spaces, health, epidemiology, Latin-America, systematic review

## Abstract

(1) Background: Increasing and improving green spaces have been suggested to enhance health and well-being through different mechanisms. Latin America is experiencing fast population and urbanization growth; with rising demand for interventions to improve public health and mitigate climate change. (2) Aim: This study aimed to review the epidemiological evidence on green spaces and health outcomes in Latin America. (3) Methods: A systematic literature review of green spaces and health outcomes was carried out for studies published in Latin America before 28 September 2020. A search strategy was designed to identify studies published in Medline via PubMed and LILACS. The search strategy included terms related to green spaces combined with keywords related to health and geographical location. No time limit for the publication was chosen. The search was limited to English, Spanish, Portuguese, and French published articles and humans’ studies. (4) Findings: This systematic review found 19 epidemiological studies in Latin America related to green spaces and health outcomes. Nine studies were conducted in Brazil, six in Mexico, three in Colombia, and one in Chile. In terms of study design, 14 were cross-sectional studies, 3 ecological, and 2 cohort studies. The population included among the studies ranged from 120 persons to 103 million. The green space definition used among studies was green density or proximity (eight studies), green presence (five studies), green spaces index (four studies), and green space visit (two studies). The health outcomes included were mental health (six studies), overweight and obesity (three studies), quality of life (three studies), mortality (two studies), cardiorespiratory disease (one study), disability (one study), falls (one study), and life expectancy (one study). Eleven studies found a positive association between green spaces and health, and eight studies found no association. (5) Conclusion: This systematic review identified 19 epidemiological studies associating green spaces and health outcomes in Latin America. Most of the evidence suggests a positive association between green spaces and health in the region. However, most of the evidence was supported by cross-sectional studies. Prioritizing longitudinal studies with harmonized exposure and outcome definitions and including vulnerable and susceptible populations is needed in the region.

## 1. Introduction

Climate change is a major human health risk worldwide, and Latin America is vulnerable to its impacts [1]. Latin American countries vary significantly in terms of their development and epidemiological profiles, suffering from infectious disease to an increased incidence of non-communicable diseases [2]. In the region, 80% of the population lives in urban environments. The urban environment has also been proposed to be a major health determinant [3]. Several interventions to mitigate climate change have been suggested, such as shifting to renewable energy sources, energy efficiency, promotion of public and active transportation, low carbon economy, forest, and biodiversity preservation, among others [4]. In recent years, green spaces in urban settings have been identified as an important climate mitigation strategy that could promote multiple health co-benefits.

Green spaces have been related to restoration, reduced stress, and increased physical activity, social capital, and ecosystem services—such as improved air quality, reduced traffic noise and heat island effects, and greater biodiversity, among others [5,6]. Regarding health outcomes, green spaces have been associated with improved mental health, immune system, metabolic system, and pregnancy outcomes, a reduction in cardiovascular diseases (CVDs), and reduced premature mortality, but the evidence still limited [7,8,9,10,11]. On the other hand, green spaces have also been related to some health risks such as exposure to allergens (e.g., pollen), pesticides, herbicides, vector-borne diseases transmitted by arthropods (e.g., Lyme disease or dengue), accidental injuries due to activities performed in greens areas, excessive exposure to ultraviolet radiation, or crime [5,12]. Four recent literature reviews have suggested that current epidemiological evidence on exposure to green spaces could lead to health benefits. [6,10,13,14]. However, many uncertainties have also been reported among those epidemiological studies.

Latin America is experiencing fast population and urbanization growth, with rising demand for interventions to improve public health and mitigate climate change [1,15]. Providing health evidence to stakeholders around green spaces in the region will help support climate mitigation strategies and public health interventions. To our knowledge, no study has summarized the available epidemiological evidence between green spaces and health outcomes in Latin America. For this reason, we aimed to review the epidemiological evidence between green spaces and health in Latin America.

## 2. Methods

This study is a systematic review of epidemiological studies on green spaces and health outcomes in Latin America. For this review, we defined Latin America as Central and South America and Spanish-speaking Caribbean countries. The study protocol was developed in accordance with the reporting guidance in the preferred reporting items for systematic reviews and meta-analyses protocols (PRISMA-P) statement and registered in the International Prospective Register of Systematic Reviews (PROSPERO-CRD42020155377).

### 2.1. Literature Search

A search strategy was designed to identify studies published in Medline via PubMed and LILACS (Latin American and Caribbean Health Sciences Literature). The search strategy included a combination of the medical subject headings (MeSH) and free-text terms for the condition of interest. No time limit for the publication was chosen. The search strategy included terms related to green spaces combined with keywords related to health and geographical location. Additionally, the search was extended to papers and reports cited in the literature but not in Medline or LILACS. The search was limited to English, Spanish, Portuguese, and French published articles and studies on humans. The last search was conducted on 28 September 2020. The results of the searches were cross-checked to eliminate duplicates.

#### Search Strategy

((green space OR greenspace * OR greenness OR greenery OR wilderness OR wild land OR natural land OR natural environment OR municipal land OR community land OR public land OR open land OR wild space OR municipal space OR natural space OR open space OR municipal park OR park OR botanic park OR park access OR urban park OR city park OR park availability OR public garden OR natural neighbourhood OR natural facilities OR vegetation natural OR belt green OR wild area OR trail green OR natural area * OR green area * OR built environment OR urban design OR recreation resource OR woodland OR forest OR pocket park OR shifrin yoku OR forest bathing OR ndvi OR normalized difference vegetation index OR healthy cities OR healthy environments)

OR

(espacio verde OR verdor OR vegetacion OR entorno natural OR ambiente natural OR espacio publico OR espacio silvestre OR espacio municipal OR espacio natural OR espacio abierto OR parque municipal OR parque OR parque botanico OR acceso al parque OR parque urbano OR parque de la ciudad OR disponibilidad del parque OR jardin OR jardin publico OR vecindario natural OR instalaciones naturales OR vegetacion natural OR cinturon verde OR area silvestre OR sendero verde OR area natural OR area verde OR entorno construido OR diseño urbano OR recurso de recreacion OR bosque OR parque de bolsillo OR shifrin yoku OR baño de bosque OR ciudades saludables OR ambientes saludables OR entornos saludables)

OR

(espaço verde OR vegetação OR ambiente natural OR espaço publico OR espaço selvagem OR espaço municipal OR espaço natural OR espaço aberto OR parque municipal OR parque OR parque botânico OR acesso ao parque OR parque urbano OR parque da cidade OR disponibilidade do parque OR jardim OR jardim publico OR bairro natural OR instalações naturais OR vegetação natural OR faixa verde OR area selvagem OR caminho verde OR area natural OR area verde OR ambiente construido OR design urbano OR instalação de recreação OR floresta OR parque de bolso OR banho florestal OR cidades saudaveis OR ambientes saudaveis))

And

(((((((“Health”[Mesh]) OR “Morbidity”[Mesh]) OR “Life Expectancy”[Mesh] OR “Quality of Life” “[Mesh])) OR ((health OR morbidity OR mortality OR life expectancy OR quality of life))))))

And

(((Latin America OR Central America OR South America OR Mexico OR Cuba OR Dominican Republic OR Haiti)) OR ((((“Latin America”[Mesh]) OR “Central America”[Mesh]) OR “South America”[Mesh]) OR “Mexico”[Mesh] OR “Cuba”[Mesh]) OR “Dominican Republic”[Mesh] OR “Haiti”[Mesh])))

And

((((((“Observational Study” [Publication Type] OR “Epidemiologic Studies”[Mesh] OR “Case-Control Studies”[Mesh] OR “Cohort Studies”[Mesh] OR “Controlled Before-After Studies”[Mesh] OR “Cross-Sectional Studies”[Mesh] OR “Historically Controlled Study”[Mesh] OR “Interrupted Time Series Analysis”[Mesh]))))

### 2.2. Study Selection

**Figure 1:**
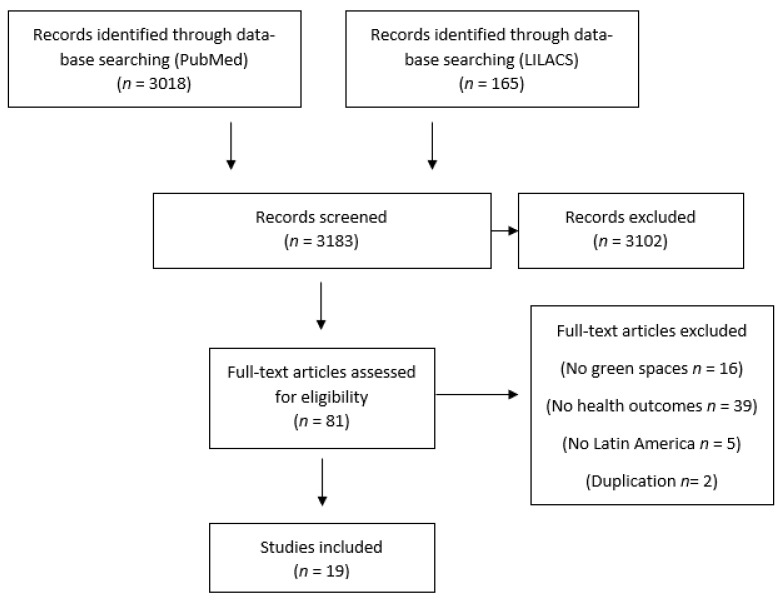
Flow chart of the selection process of epidemiological studies on green spaces and health outcomes in Latin America (end-date of search 28 September 2020).

We included epidemiological studies examining associations between green spaces and health outcomes. Inclusion criteria were: (i) the exposure of interest was measured green space, (ii) the endpoint of interest was health outcome, (iii) study in humans, (iv) studies focused in Latin America, and (v) epidemiological studies. Green space was defined as the physical environment, such as nature, vegetation, parks, and green areas. Health outcomes were defined as morbidity, mortality, life expectancy, and quality of life. In the case of quality of life, we include it as a health outcome because it provides a different dimension of health concerning the subjective measure of well-being compared to only the absence/presence of disease. When multiple published reports were from the same study, we included only the one with the most detailed information (occurred once) [16]. Two investigators (DRR and EV) independently and blindly screened the titles and abstracts to determine the articles’ inclusion (Figure 1). Eligibility criteria were applied to the full-text articles during the final selection. When discrepancies occurred (occurred 5 times), an agreement was charted out to make a final decision.

### 2.3. Data Extraction

Data extracted from each study included first author, publication year, city and country, study type, study population, green space exposure definition, health outcome, and the available adjusted risk/prevalence ratio or beta, with 95% confidence intervals (CI). A narrative synthesis of the included studies was carried out by country, study type, exposure definition, health outcomes, and population.

### 2.4. Quality Assessment

Quality of included studies was assessed using the National Institutes of Health (NIH) Quality Assessment tool for Observational Cohort and Cross-Sectional Studies [17]. This tool scores 14 items, assessing study objectives definition, study population, exposure assessment, and analyses performed. The results of such assessment considered “good” (>8 points), “fair” (6–8 points), or “poor” (<5 points) study quality appraisal.

## 3. Results

### 3.1. Description of Included Studies

The process of study identification and inclusion is shown in Figure 1. A total of 3183 studies were screened, of which 3018 studies were found in MEDLINE, and 165 in LILACS. We excluded a total of 3102 studies after independent screening titles and abstracts by two reviewers (DRR and EV) due to a lack of inclusion criteria. After a full-text review of the selected 81 studies, 16 articles were excluded because green spaces were not included, 39 were excluded because no health outcomes were included, 5 were excluded because Latin American countries or regions were not included, and 2 were excluded due to duplication. Finally, 19 studies were included in the review (Table 1).

### 3.2. Result of the Studies’ Assessment

**Figure 2:**
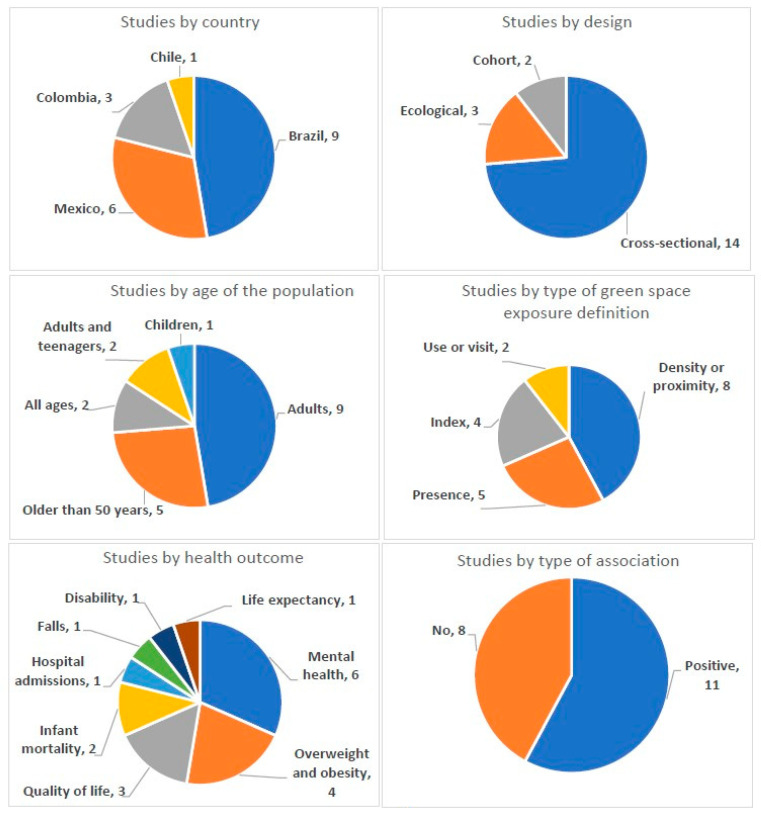
Summary of the studies characteristics and findings.

For the 19 studies included 14 were cross-sectional [16,18,19,20,21,22,23,24,25,26,27,28,29], 3 ecological studies [30,31,32], and 2 longitudinal cohorts [33,34] (Figure 2). Nine studies were conducted in Brazil [16,19,20,26,27,28,29,30,33], six in Mexico [24,25,31,32,34,35], three in Colombia [21,22,23], and one in Chile [18] (Figure 2). The studies were published between 2007 and 2019. The population ranged between 120 and 103 million individuals. In terms of age, nine studies focused on adults [16,20,22,24,25,27,29,34,35], five on individuals older than 50 years [21,26,28,33,34], two on the whole population [31,32], one included adults and teenagers [18,23], and one only included children [19] (Figure 2).

The exposure to green spaces was included using five general definitions. Eight studies used green space density or proximity [16,19,21,22,24,27,28,34], five used green presence [18,20,26,29,33], four used an index that incorporated an element such as vegetation, nature or parks [25,30,31,32], and two used green space or park visit [23,35] (Figure 2).

In terms of health outcomes, six studies focused on mental health [18,24,25,27,34,35] (Figure 2). Specifically, studies included psychiatric symptoms [18], non-psychotic mental disorders [27], depressive symptoms [24,25], depression [34], and cognitive function [35] as mental health outcomes. Four studies focused on overweight and obesity [26,29], excess weight [20], body mass index (BMI), and waist circumference [19]. Three studies focused on quality of life [21,22,23]. Two focused on infant mortality [30,32]. One study focused on the risk of hospital admissions due to cardiorespiratory diseases [16], one in falls [28], one in disability [33], and, finally, one on life expectancy [31] (Figure 2).

Overall, associations between green spaces and health outcomes were found in eleven studies [16,19,20,21,22,23,27,30,31,32,35], of which eight had a cross-sectional design [16,19,20,21,22,23,27,35] and three were ecological studies [30,31,32]. Among the cross-sectional studies the association was found between: (a) higher residential vegetation exposure, using normalized difference vegetation index (NDVI), in groups with lower risk of non-psychotic mental disorders and low-income (in a buffer 400 m from home odd ratio (OR) =0.59 (95% confidence intervals (CI)0.35–0.99), and in a buffer 1500 m from home OR =0.52 (95% CI 0.30–0.91) [27]; (b) between better quality of life and park visit with a companion (PR = 1.12 (95% CI 1.01–1.25)), and tree condition status (prevalence ratio (PR) =1.20(95% CI 1.07–1.34)) [23]; (c) between green space visits and cognitive functions (no OR was reported, but the positive association was described in the study) [35]; (d) between public park density in the neighborhood and better self-rated health (OR = 1.31(95% CI 1.01–1.71)) [21]; (e) amount of green spaces and risk of cardiorespiratory hospital admissions (no OR was reported, but the study described a significant positive association per 1 km^2^ of green areas and the reduction in two hospital admissions) [16]; (f) between home distance to parks or playground and less body mass index (BMI) (β = −2.15 (95% CI −2.53 to −1.77)) [19]; (g) between park factor, an exposure combination between park density and size, and quality of life, specifically on a positive perception about the future (OR= 1.20 (95% CI 1.10–1.14)) [22]; finally, (h) between presence of parks, squares and locations for physical activity and less excess weight (PR= 0.86 (95% CI 0.73–0.99)) [20].

Among the three ecological studies, an association was found between: (a) the sustainable development index (that included the presence of rain forest and forest coverage) and mortality in children less than five years old (β = −0.624 (95% CI no reported)) [30]; (b) between an environmental index (that included the presence of vegetation, agriculture, forestry, and grassland) and life expectancy at birth in men (β = 0.09 (95% CI 0.01–0.17)) [31]; (c) between a neighborhood environment index (that included hectares of parks per 1000 residents) and infant mortality clusters, where they found two clusters of infant mortality in those areas with high vulnerability in the environment index (no risk estimates reported, only a description in the text describing an significant association) [32]. Between the two cohort studies, no association was found [33,34]. In terms of study quality, from the 19 studies only the 2 cohort studies scored as “good” quality on the NIH quality assessment tool [33,34].

## 4. Discussion

This systematic review found 19 epidemiological studies in Latin America on green spaces and health outcomes. The majority of evidence found in Latin America reported an association between green spaces and better health outcomes related to mental health, cardiorespiratory diseases, body mass index and excess weight, quality of life, mortality, and life expectancy (11 studies). Eight studies did not find a link between green spaces and health outcomes. This review offers a comprehensive overview of the latest available evidence on green spaces and health outcomes in Latin America. To our knowledge, this is the first systematic review addressing this topic in Latin America.

Our study results are consistent with previous systematic reviews on green spaces and health outcomes worldwide [5,10]. A previous systematic review, published in 2018, identified 68 epidemiological studies related to health outcomes and health determinants, but none of the studies reported were in Latin American countries [10]. In contrast with previous reviews in the field, which combined health determinants with health outcomes, we provided a general overview of green spaces and health, focusing on morbidity, mortality, life expectancy, and quality of life. Similar to other global systematic reviews [5,10], the epidemiological evidence in Latin America on green spaces and health has been done mostly using a cross-sectional study design (14 studies). Since the outcome and exposure variables are measured at the same time, it is relatively difficult to establish causal relationships from a cross-sectional study. Cross-sectional studies are usually fast and inexpensive to conduct. They are suitable for generating hypotheses and may provide information about the prevalence of outcomes and exposures that informs other study, but do not provide evidence on causality. In addition, we found three studies using an ecological study design, which uses as a unit of observation the population or community. Despite several practical advantages of ecologic studies, there are many methodologic problems that severely limit causal inference, including ecologic and cross-level bias, problems of confounder control, within-group misclassification, lack of adequate data, temporal ambiguity, collinearity, and migration across groups. Finally, from the 19 studies found only two included a longitudinal cohort. The lack of longitudinal studies on green spaces and health in Latin America is a significant gap that limits the capacity to translate the evidence (Table 2). A previous systematic review focused on green spaces and mortality, only one longitudinal study in developing countries was found [36]. The cohort study was done in China, focused on older adults, and found an inverse association between NDVI and mortality [36]. Although the results published by Ji et al. [36] are consistent with what has been found in developed countries to date, it is unknown if variations in the use, quality, or characteristics of green spaces in Latin America could result in different impacts on health.

This systematic review also highlights the variability in exposure definitions used for green spaces in Latin America. The most common definition used was green density or proximity (8 studies), followed by green presence (5 studies), green indexes that combine one or more green definitions (4 studies), and green infrastructure visits (2 studies). Due to the variability in the exposure definition, we could not perform a quantitative summary of the studies. The use of different exposure definitions reduced the comparability between studies. Even between those that use similar definitions, such as parks or green areas, the quality, and accessibility of those parks or green areas are not defined equally, or in some cases, the information was not reported in the papers. A recent meta-analysis between green spaces and mortality combined cohort studies from seven countries in Asia, Europe, and North America that used similar exposure assessment [37]. In this meta-analysis, the cohort studies provided NDVI as an indicator for exposure, specifically levels of NDVI around homes (with buffers of 250–500 m) [37]. Similarly, NDVI exposure has been used by other study designs and health outcomes [10]. NDVI has the advantage of being freely available for any location in the globe at 30 m × 30 m resolution from satellite images, make it one of the most common green spaces definitions used among epidemiological studies [5,38]. Other common green space exposure definitions in epidemiological studies are density or percentage of green space by area, parks presence, and proximity to green space or parks [5,10]. When parks are used, epidemiological studies define the presence or absence of the park size, often reported as 0.5 hectares, 1 ha, or 2 ha. In terms of accessibility, parks or green space distance are often reported by linear distance using buffers around 250 m, 300 m, and 500 m in size [5,10]. Other indicators of green spaces have been reported, such as park usage, tree canopy, or self-reported accessibility to green space. However, based on the availability and comparability with other green space definitions, it could be beneficial that studies in the Latin American region could consider including some of those common international exposure definitions (e.g., NDVI, density or percentage of green space by area, parks presence, and proximity to green space or parks) (Table 2), in addition to specific (and newer) exposure definition then could capture different dimensions of green spaces and health interactions.

In terms of health outcomes, similarly to what was mentioned previously for the exposure definitions, differences between health outcome definitions among the Latin American studies included made the comparability, synthesis, and application of the results into health impact assessments difficult. In this review, we identified six studies on mental health. Of those, only two reported an association and were both cross-sectional studies. One study in Brazil reported a reduced risk of non-psychotic mental health disorders in low-income groups with proximity to residential vegetation (using NDVI) [27]. Another study in Mexico found an association between visiting green spaces and improved cognitive functions [35]. Four studies focused on green spaces with weight and BMI, all with a cross-sectional study design. Of those, only two found an association. One study in Brazil compared residential distance to parks or playgrounds (10 min vs. 20 min) with a reduced BMI in a low-income population [19]. Another study, also from Brazil, found that parks, squares, and locations for physical activity were associated with lower prevalence rates of excess weight [20]. Three other studies included quality of life as a health outcome, and all found an association with green spaces (all with cross-sectional study design and conducted in Colombian cities). One study reported that the “park visit with a companion” variable increase in the prevalence rate of reported improved quality of life, and also found that the “trees conditions” influence the quality of life [23]. The second one reported that park density in the neighborhood was positively associated with reporting excellent self-rated health [21]. The third one reported that an increased park factor (a combination of increased park density and size) was associated with increased probabilities to feel positive about the future [22]. Two studies focused on childhood mortality and one on life expectancy (all three with an ecological study design). In terms of mortality, one study found an association between a better sustainable development index (that combines several environmental factors, including the presence of Atlantic forest and forest coverage) with reduced mortality in children less than five years old in 37 municipalities in Brazil. A second ecological study on mortality in Mexico found that the areas in Hermosillo’s city with the highest neighborhood vulnerability index (that included lack of parks) reported two clusters of infant mortality [32]. One ecological study on life expectancy found an association among an environmental index (that included vegetation, agricultural, forestry, and grassland indicators) at the national level and better life expectancy among males in Mexico [31]. One cross-sectional study found an association between the amount of green areas in the Federal District’s urban area in Brazil and a reduced number of cardiorespiratory hospital admission [16]. Finally, green spaces were studied in relationship with disabilities [33] and with falls [28], but an association was not found in either study. To increase comparability and evidence translation, it is recommended that studies including specific diagnoses report clear disease definitions, especially using the International Classification of Disease (ICD-10 or 11) diagnostic tool. It is also important to report the clinical tool used to define symptoms or mental health and cognitive outcomes in studies reporting those. Additionally, if possible, identify which outcome definitions have been used by other epidemiological studies and include a similar health outcome to increase comparability (Table 2). Additionally, we found a lack of studies focusing on susceptible (e.g., children, those with comorbidities) and vulnerable (e.g., low-income, socially deprived) population. This is of special interest in terms of health equity and environmental justice. In this regard, health evidence on these populations could help prioritize land use and built environment policies to reduce environmental health inequalities.

### 4.1. Limitations of the Available Evidence

As with any systematic review, this study has several limitations. Publication bias is one of the most important limitations of all systematic reviews. To reduce the publication bias, the inclusion criteria were not limited by time or population. We tested and included a search strategy with free text and MeSH terms. We also performed the search strategy for PubMed and in LILACS, where it is expected that regional publications can be concentrated. Another limitation was the lack of similar definitions of exposure and outcomes, reducing the possibilities to translate the systematic review into a meta-analysis. This review selected only those epidemiological studies that reported morbidity, mortality, life expectancy, or quality of life. The current epidemiological evidence provides a large body of studies focusing on health determinants related to green spaces (e.g., physical activity, air quality, and humidity, among others) that were beyond the scope of this review. We favored health outcomes that could be more easily translated into public health practices, although we acknowledge that health determinants could explain the pathways on how green spaces affect health. Thus, this systematic review should be considered a complementary tool to understand the universe of evidence available on green spaces and health in the Latin American region.

### 4.2. Recommendations

We included in Table 2 a list of recommendations for studies on green spaces and health outcomes in the Latin America region. These recommendations are based on the review findings and will increase the comparability and translation of the evidence in the region. When epidemiological studies on green spaces and health are planned in the Latin American region, a consideration to include at least one similar definition of the exposure and health outcome used in previous international studies will increase comparability among studies. Additionally, a clear definition of the health outcome is crucial for translation and comparability purposes. None of the 19 studies found described the International Classification of Disease code (ICD) of the health outcome. This description is often absent in epidemiological studies and limits comparability and translation. In addition, although the Latin America region comprises 20 countries, this review only found epidemiological evidence in three countries (Brazil, Mexico, and Colombia). Further support for environmental health research, especially green spaces and health, is needed among Latin American countries. Furthermore, more collaboration among countries and the development of multinational studies, with similar protocols, taking advantage of those (freely) available green space exposure data will increase the evidence in the region. The quality assessment also highlighted the importance of increasing studies with “good” quality. In the review, only two studies (from the 19 included) were assessed with good quality, mainly due to the type of study design. Table 2 also includes a description of the main gaps and key areas to be considered in future studies in the region. We are especially concerned about the lack of longitudinal studies, focus on vulnerable populations (e.g., children or pregnant women), and a clear and comparable definition of exposures and outcomes across epidemiological studies.

## 5. Conclusions

This systematic review found epidemiological evidence to support green spaces to improve health outcomes in Latin America. Cross-sectional design is the most common type of study design used on green spaces and health outcomes in the Latin American region. More studies using longitudinal approaches with harmonized exposure and outcome definitions, including vulnerable and susceptible populations, are needed in the region.

## Figures and Tables

**Table 1:** Studies included in the literature review.

Author (Year)	Geographical Location	Study Type	Study Population	Exposure Type	Health Outcome	Risk Estimate	Lower CI	Upper CI	NIH QAT Score
Araya R, et al. 2007	Santiago, Chile	CS	3870 adults (16–64 y)	Green areas (public green areas, trees, size of trees, and green areas in sidewalks)	Psychiatric symptoms (Revised Clinical Interview Schedule total score)	β = −0.01	−0.09	0.06	Fair
Araujo C, et al. 2018	Florianopolis, Brazil	CS	1197 older adults (≥60 y)	Presence of recreational green areas	Obesity	Females OR = 1.02	0.70	1.50	Fair
						Males OR = 1.14	0.71	1.84	
Barreto P, et al. 2019	Rio de Janeiro, Brazil	CS	2584 adults (25–75 y)	NDVI (buffer 200, 400, 1500 m)	Non-psychotic mental disorders (General health questionnaire)	Low-income group (200 m buffer) OR = 0.72	0.42	1.24	Fair
						Intermediate income group (200 m buffer) OR = 0.88	0.61	1.28	
						High-income group (200 m buffer) OR = 0.98	0.67	1.44	
						Low-income group (400 m buffer) OR = 0.59	0.35	0.99	
						Intermediate income group (400 m buffer) OR = 0.77	0.52	1.14	
						High-income group (400 m buffer) OR = 0.92	0.63	1.35	
						Low-income group (1500 m buffer) OR = 0.52	0.3	0.91	
						Intermediate income group (1500 m buffer) OR = 0.95	0.66	1.38	
						High-income group (1500 m buffer) OR = 0.79	0.53	1.18	
Bojorquez I, et al. 2018	Tijuana, Mexico	CS	2345 adult woman (18–65 y)	Park coverage, with a size of 500 m2 (in 400 m and 800 m buffer), and vegetation coverage (in 400 m and 800 m buffer)	Depressive symptoms (Center for Epidemiologic Studies-Depression Scale)	Park coverage in 400 m buffer β = −0.01	−0.01	0	Fair
						Park coverage in 800 m buffer β = −0.00	−0.01	0	
						Vegetation coverage in 400 m β = −0.03	−0.15	0.09	
						Vegetation coverage in 800 m β = 0.04	−0.12	0.2	
Bueno R, et al. 2013	Curitiba, Brazil	E	Children <5 y in 37 municipalities	Sustainable development index (remaining of the Atlantic rainforest, proportion of forest coverage)	Mortality in children	β = −0.624			Fair
Camargo D, et al. 2017	Bucaramanga, Colombia	CS	1392 park users (12–86 y)	Park visit or perception (visit park with a companion, active use of the park, tree conditions)	Quality of life (EUROHIS-QOL 8 questionnaire)	Visit the park with a companion PR = 1.12	1.01	1.25	Fair
						Active use of the park PR = 1.14	1	1.3	
						Tree conditions status PR = 1.20	1.07	1.34	
Danielewicz A, et al. 2018	Florianopolis, Brazil	Cohort	1196 older adults (≥60 y)	Green areas	Incidence in disability basic (ADL) and instrumental (IADL) activities of Daly living (Multidimensional functional Assessment Questionnaire)	ADL OR = 0.98	0.68	1.4	Good
						IADL OR = 0.92	0.62	1.35	
do Nascimento, et al. 2017	Sao Paulo, Brazil	CS	1345 older adults (≥60 y)	Green area (m3/resident) Low vs. High	Falls (indoor, outdoor)	Indoor falls PR = 0.92	0.64	1.31	Fair
						Outdoor falls PR = 0.79	0.52	1.21	
Duarte-Tagles H, et al. 2015	Mexico	CS	45,242 adults (>19 y)	Biodiversity index	Depressive symptoms	OR = 1.05	0.86	1.29	Fair
Fernandez-Nino J, 2019	Mexico	Cohort	996 adults (>50 y)	Total length of street space with trees per 100 m	Depression	Overall OR = 1	0.99	1	Good
						Urban OR = 1	0.99	1	
						Rural OR = 0.56	0.45	6.8	
Idrovo A, et al. 2011	Mexico	E	103 million inhabitants (whole country population)	Environmental index composed of 50 factors (including vegetation, agricultural, forestry, and grassland)	Life expectancy at birth	Total population β = 0.07	0	0.14	Fair
						Men β = 0.09	0.01	0.17	
						Women β = 0.06	0	0.12	
Lara-Valencia F, et al. 2012	Hermosillo, Mexico	E	784,322 inhabitants (whole city population)	Neighborhood environment index (include hectares of parks per 1000 residents)	Infant mortality clusters	Two city clusters of infant mortality found in areas with high vulnerability in the environmental index	NR	NR	Fair
Martinez-Soto J, et al. 2014	Mexico City, Mexico	CS	120 persons (17–79 y)	Urban nature (potted plants, green space visits)	Cognitive functions	Potted plants and green space visits have positive impacts on cognitive functions	NR	NR	Fair
Mendes L, et al. 2013	Belo Horizonte, Brazil	CS	3404 adults (>18 y)	Park/ public squares/ places for practicing physical activity	Overweight/ obesity	PR = 0.99	0.72	1.37	Fair
Parra D, et al. 2010	Bogota, Colombia	CS	1966 older adults (≥60 y)	Public park density of the neighborhood (% m2 of a public park)	Excellent Self-rated health (Health-related Quality of Life, short form−8)	OR = 1.31	1.01	1.71	Fair
Requia W, et al. 2016	Federal District, Brazil	CS	7269 hospital admissions	Amount of green area intra-urban (m2)	Risk of cardiorespiratory hospital admissions	1 km^2^ increase in green areas was associated with a reduction in two hospital admissions	NA	NA	Fair
Rossi C, et al. 2018	Florianopolis, Brazil	CS	2152 school children (7–14 y)	Home distance to park/playgrounds and use of parks	Body mass index (BMI), waist circumference (WC)	Distance to parks/playground (10 min vs. >20 min) in low income population BMI β = −2.15	−2.53	−1.77	Fair
						Distance to parks/playground (10 min vs. >20 min) in high-income population BMI β = 1.11	−0.12	2.34	
						Distance to parks/playground (10 min vs. >20 min) in low-income population WC in cm β = −0.07	−0.27	0.12	
						Use parks/playgrounds in low-income population WC in cm β = 0.008	−0.11	0.13	
						Use parks/playgrounds in medium-income population WC in cm β = −0.02	−0.1	0.15	
						Use parks/playgrounds in high-income population WC in cm β = −0.23	−0.48	0.03	
						Use parks/playgrounds in low-income population BMI β = 0.61	−0.79	2	
						Use parks/playgrounds in medium-income population BMI β = −0.45	−2.01	1.4	
Sarmiento O, et al. 2010	Bogota, Colombia	CS	1334 adults (median 38 y)	Parks factor (park density and size)	Health-related quality of life (WHOQOL-BREF instrument)	Perceived health status OR = 1.2	1	1.3	Fair
						Positive about the future OR = 1.2	1.1	1.14	
Velasquez-Melendez G, et al. 2013	Belo Horizonte, Brazil	CS	3425 adults (≥18 y)	Presence of parks, squares, and locations for physical activity	Excess weight	PR = 0.86	0.73	0.99	Fair

CI: Confidence intervals; NIH QAT: quality assessment tool: National Institutes of Health Quality Assessment tool for observational cohort and cross-sectional studies; CS: cross-sectional studies; E: ecological studies; OR: odds ratio; PR: prevalence rate; NDVI; normalize difference vegetation index; ADL: Incidence in disability basic: IADL: instrumental activities of daily living BMI: body mass index; WC: waist circumference; WHOQOL-BREF: World Health Organization Quality of Life self-report questionnaire.

**Table 2:** Recommendations for future research on green spaces and health in Latin America.

Research Area	Recommendations
	Report a clear definition of green spaces.
Exposure assessment	Identify and use at least one common international definition of green spaces to increase comparability among epidemiological studies. NDVI, density or percentage of green space by area, park presence, proximity to green space or parks. If parks are used, report size (i.e., >0.5 ha, >1 ha, or 2 ha). If accessibility/proximity is used, report distance (i.e., 250 m, 300 m, or 500 m).
Study design	Favor cohort studies. Favor quasi-experimental studies. Favor case-crossover studies. Support exposure assessment.
Population	Favor vulnerable or susceptible populations (e.g., children, pregnant women, people with comorbidities, and disadvantaged populations). Support research in all Latin American countries. Support research of multinational studies.
Outcomes	Report a clear definition of the health outcome (e.g., diagnosis, ICD-10/11, and a clinical tool used to define health outcome).
	Identify and use at least one common definition of health outcome used in previous studies. This will increase the comparability, evidence synthesis, and application of the results into health impact assessments.
Analysis	Include analysis by age, sex, socioeconomic status, or any other subpopulation available. Include confidence intervals and *p* values.
	Include a dose–response function, if possible.

NDVI: normalize difference vegetation index; ICD: International Classification of Diseases.
